# Supplementary material for: Evaluation of a Novel Chromogenic Medium for the Detection of Pseudomonas aeruginosa in Respiratory Samples from Patients with Cystic Fibrosis
Source: Microorganisms. 2022 May 10;10(5):1004. doi: 10.3390/microorganisms10051004 (PMC9144526; doi:10.3390/microorganisms10051004)
Supplement: Supplementary file 1 [file microorganisms-10-01004-s001.zip › microorganisms-1668437-supplementary.pdf]

microorganisms

Supplementary information

Synthesis of ß-alanyl-ANDF (N-(β-alanyl)-2-amino-7-nitro-9,9-dimethylfluorene TFA salt

Analytical methods

NMR Spectroscopy was performed on JEOL ELS400 Delta Spectrometer at frequencies of 400 MHz for ^{1}H NMR and 101 MHz for ^{13}C NMR. Chemical shifts were recorded as parts per million (ppm) with tetramethylsilane (TMS) as the internal standard. Deuterated dimethyl sulfoxide (DMSO-d6) was used as a solvent. The multiplicities of signals were recorded as singlet (s), doublet (d), triplet (t) and multiplet (m). Ar indicates aromatic protons/carbons. High Resolution Mass Spectrometry (HRMS) was obtained from the EPSRC National Mass Spectrometry Service, Swansea University using a LTQ Orbitrap XL 1 spectrometer using nano-electrospray ionisation (NSI). Infra-red Spectra were recorded on a SensIR Technologies Durascope diamond anvil cell mounted on a Perkin-Elmer Paragon 1000FR-IR Spectrometer. Column Chromatography was performed with Fisher Scientific silica gel 60 Å (35-70 µm).

Synthetic methodology

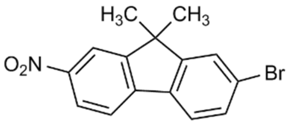

1

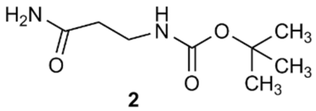

2

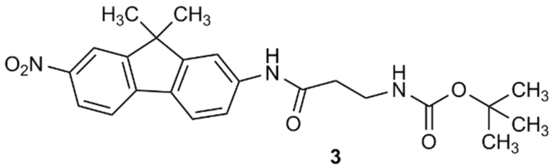

3

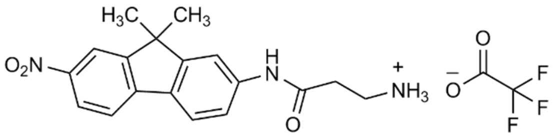

4

Substrate **4** was synthesised in a two step sequence from the starting materials **1** and **2**. A palladium catalysed cross-coupling reaction between these two starting materials afforded compound **3** from which the benzyloxycarbonyl group was removed using trifluoroacetic acid affording the required substrate **4**. 2-Bromo-7-nitro-9,9-dimethylfluorene **1** was prepared by bromination of commercially available 2-nitro-9,9-dimethylfluorene [1]. *N*-Benzyloxycarbonyl-3-aminopropanamide **2** was synthesised from commercially available *N*-benzyloxycarbonyl-β-alanine [2].

N-(N-benzyloxycarbonyl-β-alanyl)-2-amino-7-nitro-9,9-dimethylfluorene ***3***

A mixture of 2-bromo-7-nitro-9,9-dimethylfluorene **1** (3 g, 9.46 mmol, 1.1 equiv.), Cs_2CO_3 (392 mg, 12.40 mmol, 1.4 equiv.), amide **2** (1.55 g, 8.6 mmol, 1 equiv.) and Xantphos (298 mg, 0.5 mmol, 0.06 equiv.) in dimethylformamide (40 mL) was stirred for 20 minutes at room temperature under a nitrogen atmosphere. $Pd_2(dba)_3$ (58 mg, 0.02 equiv.) was then added and the mixture and heated to 100 ^oC for 24 hours. The mixture was allowed to cool to room temperature, poured into water (60 mL) and the precipitate was collected by filtration and washed with water. The precipitate was suspended in acetone and the resulting mixture was filtered through celite. The celite was washed with acetone and the combined acetone fractions were concentrated under reduced pressure to give crude product. Purification by column chromatography over silica gel (CH_2Cl_2:ethyl acetate 2:1 R_f = 0.45) afforded the desired product **3** as yellow crystals (1.4 g, 3.27mmol, 38 %). HRMS (+NSI) Found 426.2027 (calcd. 426.2027 for $C_{23}H_{28}N_3O_5$; $[M+H]^+$). IR ν_{max} (cm^{-1}): 3309 (N-H), 2971, 1698 (C=O), 1680 (C=O), 1504 and 1334. ^{1}H-NMR (400 MHz, DMSO-d_6). δ: 10.17 (1H, s, NH), 8.41 (1H, d, J = 1.8 Hz, ArH), 8.20 (1H, dd, J = 8.2 and 1.8 Hz, ArH), 7.95 (1H, d, J = 8.2 Hz, ArH), 7.91-7.88 (2H, m, ArH), 7.52 (1H, d, J = 9.2 Hz, ArH), 6.88 (1H, t, J = 5.5 Hz, NH), 3.30 (2H, overlapped by solvent water peak, CH_2), 3.18 (2H, t, J = 6.4 Hz, CH_2), 1.44 (6H, s, $C(CH_3)_2$), 1.33 (9H, s, $C(CH_3)_3$).

N-(β-alanyl)-2-amino-7-nitro-9,9-dimethylfluorene TFA salt ***4***

To stirred *N*-(benzyloxycarbonyl-β-alanyl)-2-amino-7-nitro-9,9-dimethylfluorene **3** (80 mg, 0.19 mmol) in CH_2Cl_2 (4 mL) was added trifluoroacetic acid (1 mL) dropwise. The mixture was stirred at room temperature for 4 hours and then evaporated. The resulting oil was triturated with ether giving the desired product as a yellow solid (79 mg, 0.18 mmol, 95 %). IR ν_{max} (cm^{-1}): 2967, 1668 (C=O), 1522, 1332. HRMS (+NSI) Found 326.1497 (calcd. 326.1499 for $C_{18}H_{20}N_3O_3$; $[M]^+$). ^{1}H-NMR (400 MHz, 8DMSO-d_6). δ: 10.40 (1H, s, NH), 8.43 (1H, s, ArH), 8.22 (1H, d, J = 8.2 Hz, ArH), 7.91-7.93 (3H, m, ArH), 7.43 (3H, s, NH_3^+), 7.52 (1H, d, J = 8.7 Hz, ArH), 3.08 (2H, m, CH_2), 2.70 (2H, t, J = 6.4, CH_2), 1.44 (6H, s, $C(CH_3)_2$). ^{13}C-NMR (101 MHz, DMSO-d_6). δ: 169 (C=O), 157 (Ar-C), 155 (Ar-C), 147 (Ar-C), 146 (Ar-C), 141 (Ar-C), 132 (Ar-C), 124 (Ar-C), 123 (Ar-C), 121 (Ar-C), 119 (Ar-C), 119 (Ar-C), 114 (Ar-C), 47.5 ($\underline{C}$-$(CH_3)_2$), 35 (CH_2), 34 (CH_2), 27 (CH_3 x2).

Supplementary References

[1] Hallas, G.; Hepworth, J.D.; Waring D.R. Extended conjugation in di- and tri-arylmethanes. Part I. Electronic absorption spectra of 9,9-dimethylfluorene analogues of crystal violet and malachite green *J. Chem. Soc. (B)*, **1970**, 975-979, https://doi.org/10.1039/J29700000979

[2] Shin, K.J.; Yoo, K.H.; Kim, D.J.; Park, S.W.; Ko, B.S.; Lee, S.J.; Huh, J.D.; Park, S.Y. Synthesis and biological properties of new 1β-methylcarbapenems. *Bioorg. Med. Chem. Lett*. **1998**, *8*, 1607-1612, https://doi.org/10.1016/S0960-894X(98)00270-4.

Acknowledgements

We thank the EPSRC UK National Mass Spectrometry Facility at Swansea University, UK, for high resolution mass spectra.
